# Mediation of the effect of prenatal maternal smoking on time to natural menopause in daughters by birthweight-for-gestational-age z-score and breastfeeding duration: analysis of two UK birth cohorts born in 1958 and 1970

**DOI:** 10.1186/s12905-025-03556-y

**Published:** 2025-01-23

**Authors:** Darina Peycheva, Leah Li, Mary Fewtrell, Richard Silverwood, Rebecca Hardy

**Affiliations:** 1 Institute of Epidemiology and Health Care, University College London (UCL), London, WC1E 7HB UK; 2 GOS Institute of Child Health, UCL, London, UK; 3 Social Research Institute, UCL, London, UK; 4 School of Sport, Exercise and Health Sciences, Loughborough University, Loughborough, UK

**Keywords:** Smoking during pregnancy, Menopause, Birthweight, Breastfeeding, Causal mediation analysis, Inverse odds weighting, Birth cohort, Longitudinal

## Abstract

**Background:**

Prenatal maternal smoking, lower birthweight, and shorter breastfeeding duration have all been associated with an earlier age at menopause in daughters. We estimated the extent to which birthweight-for-gestational-age z-score and breastfeeding duration mediate the effect of prenatal maternal smoking on time to natural menopause in daughters.

**Methods:**

Using pooled data from two prospective birth cohort studies – the 1970 British Cohort Study (*n* = 3,878) followed-up to age 46 years and the 1958 National Child Development Study (*n* = 4,822) followed-up to age 50 years – we perform mediation analysis with inverse odds weighting implemented in Cox proportional-hazards models.

**Results:**

Prenatal maternal smoking was associated with lower birthweight z-scores [β: -0.29; 95% CI -0.34, -0.24] and reduced breastfeeding duration [RRR_< 1month_: 0.90; 95% CI 0.79, 1.02; RRR_≥ 1 month_: 0.66; 95% CI 0.59, 0.73 relative to women who were never breastfed]. Greater z-score for birthweight [HR: 0.96; 95% CI 0.91, 1.01] and longer breastfeeding duration [HR_≥ 1 month_: 0.84; 95% CI 0.74, 0.96] were associated with lower hazards for earlier age at natural menopause. The total effect of prenatal maternal smoking on the time to natural menopause in daughters was estimated as a HR of 1.13 [95% CI 1.02, 1.24]. Birthweight z-score and breastfeeding duration jointly explained an estimated 14% of the total effect [HR_NIE_: 1.02; 95% CI 0.99, 1.05].

**Conclusions:**

The consequences of smoking during pregnancy on the earlier experience of natural menopause in daughters may partly be offset by intrauterine growth and longer breastfeeding duration to the extent that they mediate the risk of earlier menopause. However, since the extent of mediation by birthweight z-score and breastfeeding duration is small, other factors, including the direct effect of maternal smoking in utero, may play a more important role.

**Supplementary Information:**

The online version contains supplementary material available at 10.1186/s12905-025-03556-y.

## Background

Natural menopause occurs when the ovarian follicle pool is exhausted. Reaching its peak at around 20 weeks of fetal life, the pool of ovarian follicles declines gradually thereafter. The individual differences in the initial number of ovarian follicles and the rate of ovarian follicle loss are considered to determine menopausal age [1]. Among white women from high-income countries natural menopause occurs on average between 50 and 52 years, though around 10% experience menopause before the age of 45 years. Earlier age at natural menopause has important health implications such as increased risk of type 2 diabetes, cardiovascular disease, osteoporosis, and all-cause mortality [2]. 

Clinical studies have shown that toxic agents, including cigarette smoke, may contribute to ovarian follicle loss before birth and alter the reproductive span of female offspring [3]. In epidemiological studies, the effect of in utero exposure to cigarette smoke on the timing of menopause in female offspring is contested [4–9]. Some studies show no effect, however, they all present estimates adjusted for mediators (variables that may be on the causal pathway), such as birth weight, breastfeeding, and other events in the daughter’s early and later life [5, 6, 8]. Adjustment for mediators will tend to underestimate the overall effect of maternal smoking during pregnancy and under certain assumptions, the adjusted estimate will represent the part of the effect that is not mediated by the intermediate variable(s) [10]. Studies that have not adjusted for mediators show that women prenatally exposed to maternal cigarette smoke may undergo menopause at an earlier age [4, 7, 9]. Understanding the role of mediators in the effect of in utero exposure to cigarette smoke on the timing of menopause in female offspring can help identify pathways to offset the potentially deleterious effect of such exposure [11]. 

Fetal growth and breastfeeding are two potential mediators between maternal smoking during pregnancy and earlier age at menopause in daughters (that have previously been adjusted for as if they were confounders). Previous research has suggested that low birth weight and not being breastfed may be associated with the timing of menopause, although findings are not entirely consistent [2, 4, 12–15]. 

Restricted fetal growth is the most consistent effect of prenatal cigarette smoke exposure. Nicotine interacts with receptors in placental vasculature resulting in decreased placental blood flow and fetal vasoconstriction which leads to disruption of the delivery of oxygen and nutrients to the fetus. This reduced blood flow leads to fetal malnutrition and is thought to be a causal mechanism for the effects of prenatal cigarette smoke exposure on poor fetal growth [16]. Birth weight deficits in infants prenatally exposed to cigarette smoke range from 200 to 327 g, depending on the nicotine dose; it is estimated that 20% of low birthweight and small for gestational age infants are attributable to prenatal exposure to cigarette smoking [17, 18]. 

Maternal smoking during pregnancy also affects lactogenesis and lactation. Women who smoke during pregnancy are less likely to initiate breastfeeding and tend to breastfeed for shorter periods [20–22]. Smoking in pregnancy reduces prolactin concentration (an important mediator of normal lactogenesis) which has implications for lactation [22, 23]. It has been proposed that a prolactin measurement between the 35th and 38th week of pregnancy could be a good predictor of lactational performance [24]. In addition, low birthweight is associated with delays in (or failure of) early breastfeeding initiation (within the first hours of birth) and reduced duration of exclusive breastfeeding [19]. 

This study estimates the extent to which the effect of maternal smoking during pregnancy on time to natural menopause in daughters is mediated by birthweight-for-gestational-age z-score (a marker of fetal growth rate, hereafter birthweight z-score) and breastfeeding duration.

## Methods

### Study population

We used data from two ongoing prospective British birth cohort studies. The 1970 British Cohort Study (BCS70) follows the lives of 8,655 women (18,037 people) born in a single week in March 1970 [28–30]. Since birth, study members and/or their parents have been interviewed 10 times from infancy through childhood and into adulthood. This study used data collected at birth, and ages 5, 42 and 46 [30]. The 1958 National Child Development Study (NCDS) follows the lives of 8,959 women (18,558 people) born in a single week in March 1958 [31]. Since birth, study members and/or their parents have been interviewed 11 times from infancy through childhood and into adulthood. This study used information collected at birth and ages 7, 44, and 50 [32]. Data from both studies are publicly available via the UK Data Service (UKDS) [30, 32]. 

### Outcome: age at natural menopause

Information on menstrual irregularity, month and year of last menstrual period, any surgery to remove the uterus or both ovaries, and use of hormonal therapy (HT) was collected at age 42 and 46 surveys in BCS70 and age 44 and 50 surveys in NCDS. Of the 8,655 women in BCS70 at birth, 5,117 participated at the age 42 follow-up survey (of 6,600 eligible for interview) and 4,427 participated at the subsequent age 46 survey (of 6,171 eligible). Of the 8,959 women in NCDS at birth, 4,712 participated at the age 44/45 follow-up survey (of 6,606 eligible for interview) and 4,968 women took part at the subsequent age 50 survey (of 6,139 eligible) (Figure S1, Supplementary material). Natural menopause, taken as the date of the final menstrual period (FMP), was defined retrospectively after 12 consecutive months of amenorrhea not due to surgery or other medical treatment [1]. Peri-menopausal women were those with 3 to 11 months of amenorrhea or whose periods became less regular in the absence of amenorrhea. Pre-menopausal women reported menstruation within the last 3 months. Women who provided sufficient information to determine whether they were premenopausal, perimenopausal or had undergone natural or surgical menopause, or started HT before their FMP were included in this analysis: 3,878 in BCS70 and 4,822 in NCDS. Women whose periods stopped for other reasons (e.g. pregnancy, contraceptives, chemotherapy) (274 in BCS70 and 201 in NCDS) or there was no sufficient information to determine their menopause status (384 in BCS70 and 75 in NCDS), were excluded (Table S1, Supplementary material).

### Exposure: maternal cigarette smoking during pregnancy

At birth mothers of cohort children in both studies were asked whether they smoked during the pregnancy. Maternal smoking during pregnancy was categorised as non-smokers (women who never smoked or stopped before becoming pregnant) and smokers (women who smoked during part or throughout the whole pregnancy).

### Mediators: birthweight-for-gestational-age z-score and breastfeeding duration

Information on birthweight and gestational age in both studies was recorded by a midwife at cohort member’s birth. In each cohort separately, birthweight (in kilograms), adjusted for gestational age (in weeks), was transformed to standard deviation scores (z-scores), using the LMS method [33]. Z-scores, calculated according to the British 1990 Growth Reference [34], were obtained using the *egen zanthro()* function in Stata [35]. 

At the first major survey following the birth sweep, mothers in both studies were asked if the cohort child was breastfed partly or wholly even for a few days. The available data was categorised as never breastfed, breastfed for less than 1 month, and breastfed for 1 month or longer.

### Potential confounding variables: maternal education, maternal age and father’s social class at birth, and parity

**Fig. 1:**
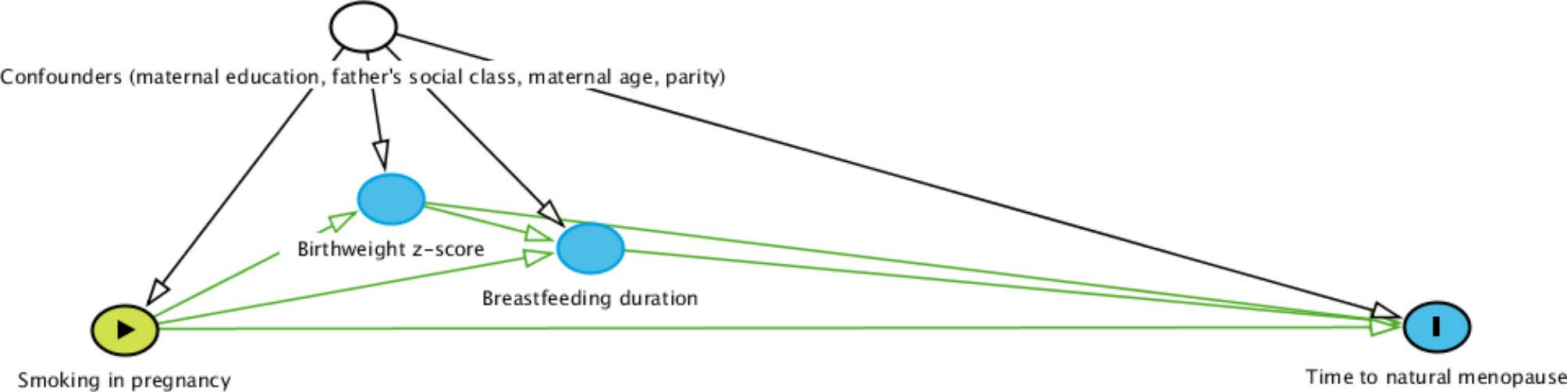
A causal diagram of the association between maternal smoking in pregnancy and time to natural menopause in daughters

To control for confounders of the exposure, mediators, and outcome (Fig. 1), we included the following preexposure characteristics: whether the mother remained in school after minimum school leaving age of 15 years (yes, no); father’s social class at birth (non-manual, manual, no father figure), maternal age at birth (years), and parity (number). The choice of confounders was based on the literature and previous research by our research group [4, 25–27]. 

### Statistical analysis

Descriptive statistics for all variables included in the analysis for each cohort separately (means and standard deviations (SDs) for continuous variables, percentages for categorical variables) for both the imputed sample (see ‘Missing data’ section) and sample with complete cases are presented in Table 1.

We assessed the associations between maternal smoking during pregnancy and birthweight z-scores and breastfeeding duration using linear and multinomial regression models and assessed the associations between birthweight z-scores and breastfeeding duration and time of natural menopause using Cox proportional hazards models (Table 2). Follow-up time in the Cox proportional hazards models was in years since age 11 (average age of puberty onset in girls) until the earliest of natural menopause, surgery or start of HT (before natural menopause), or end of study period. Follow-up was treated as censored if the event was not natural menopause. Adjusted linear regression coefficients, relative risk ratios (RRRs) and hazard ratios (HRs), and corresponding 95% confidence intervals (CIs), for both pooled and study specific analyses of multiply imputed samples are presented in Table 2. The pooled analysis incorporated a dummy variable identifying the cohort (NCDS or BCS70). Proportional hazard assessments confirmed the hazard ratio’s consistency over time, validating the proportionality assumption.

We then decomposed the total effect (TE) of maternal cigarette smoking during pregnancy on the timing of natural menopause in daughters into natural direct effects (NDE) and natural indirect effects (NIE) through birthweight z-scores and breastfeeding duration (Table 3). We used sequential causal multiple mediator analysis with inverse odds weighting (IOW) [36, 37], implemented in Cox proportional-hazards models. The odds were obtained using logistic regression model for the exposure given the mediators and confounding variables. The weights were computed by taking the inverse of the predicted odds for each observation in the exposed group; the unexposed group was assigned an IOW of 1. We estimated the TE using an unweighted Cox proportional hazards model of the outcome conditional on the exposure and confounding variables. We estimated the NDE via a weighted Cox proportional hazards model of the outcome conditional on the exposure and confounding variables, using the IOW. We calculated the NIE via the mediators by subtracting the NDE from the TE and 200 bootstrap replications were used to derive bias-corrected CIs for TE, NDE and NIE. Pooled and study specific results from multiply imputed samples, adjusted for confounding variables, are presented in Table 3. In secondary analysis we restrict the follow-up period in the pooled sample until age 46 years (the follow-up period in the younger BCS70 cohort) to allow better comparison between the cohorts in terms of follow-up time. In this analysis NCDS women were censored at age 46 (Table S6, Supplementary material).

Results from complete case analyses are presented in Supplementary material (Tables S3 and S4).

### Missing data

Missing data in the dates of menopause, surgery, or HT ranged between 2.7 and 20.1% in BCS70 and 3.0 to 22.0% in NCDS (Table S2, Supplementary material). For all events, if the year was available but the month was missing – the missing month was replaced with mid-year (July). For natural menopause, if neither month nor year was available – the missing date was replaced with the date of interview minus 12 months. For surgery or HT, if neither month nor year was available – the missing date was replaced with the date of interview.

We used multiple imputation (MI) with chained equations, performed in each cohort separately, to address the limitation of performing this analysis on a significantly reduced analytical sample due to attrition in a follow-up period spanning decades [38]. The imputation model included the exposure, all mediators, and confounders, as well as the outcome; though missing values were only imputed on exposure, mediators, and confounders [39]. The proportion of missing observations for each variable ranged between 4.8 to 18.8% in BCS70 and 5.2 to 17.3% in NCDS (Table 1). We created 20 imputed datasets; the estimates from each imputed dataset were combined to obtain overall estimates using Rubin’s rules [40]. 

All analyses were conducted using Stata 17.

## Results

The study sample comprised 8,700 women: 3,878 women in the BCS70 cohort and 4,822 women in the NCDS cohort. By the follow-up of BCS70 at age 46, 8.9% of women had experienced natural menopause, and by the follow-up of NCDS at age 50, 24.4% had undergone natural menopause. 44.3% of women in BCS70, and 41.7% in NCDS had mothers who smoked at any time during the pregnancy. Z-scores for birthweight were lower in NCDS [mean − 0.36 (SD 1.10)] compared to BCS70 [mean − 0.21 (SD 1.11)]. 40.4% of women in BCS70 and 69.5% in NCDS received breastmilk - partly or wholly even for a few days (Table 1). In both cohorts, maternal sociodemographic factors were associated with the exposure, mediators, and outcome (Table S5).

**Table 1:** Characteristics of the study sample(s)

Sample characteristic	BCS70 (*n* = 3,878, followed-up to age 46 years)			NCDS (*n* = 4,822, followed-up to age 50 years)		
	N	% complete cases	% imputed sample	N	% complete cases	% imputed sample
**Natural menopause**						
No	3,533	91.10		3,645	75.59	
Surgical menopause	331	8.54		534	11.07	
HT before FMP	174	4.49		542	11.24	
Pre- and peri- menopause	3,028	78.07		2,569	53.28	
Yes	345	8.90		1,177	24.41	
**Smoking in pregnancy**						
No	2,001	55.66	55.69	2,632	58.29	58.24
Yes (incl. stopped during pregnancy)	1,594	44.34	44.31	1,883	41.71	41.76
*Missing*	*283*	*7.30*		*307*	*6.37*	
**Birthweight (kg) (mean**,** SD)**	3,607	3.26 (0.50)		4,423	3.26 (0.51)	
*Missing*	*180*	*4.75*		*399*	*8.27*	
**Gestational age (weeks) (mean**,** SD)**^1^	3,392	39.78		4,117	40.16 (1.73)	
*Missing*	*395*	*10.43*		*705*	*14.62*	
**Birthweight z-score (mean**,** SD)**	3,379	-0.21 (1.11)	-0.21 (1.19)	3,987	-0.36 (1.10)	-0.37 (1.20)
*Missing*	*499*	*12.87*		*835*	*17.32*	
**Breastfeeding**						
Never	1,877	59.63	59.74	1,285	30.48	30.51
Up to 1 month	507	16.11	16.14	1,031	24.45	24.55
More than 1 month	764	24.27	24.11	1,900	45.07	44.94
*Missing*	*730*	*18.82*		606	*12.57*	
**In school after age 15**						
Yes	1,342	37.47	37.42	1,229	26.96	27.01
No	2,240	62.53	62.58	3,330	73.04	72.99
*Missing*	*296*	*7.63*		*263*	*5*,*45*	
**Social class at birth**						
Non-manual	1,131	31.47	31.42	1,248	27.30	27.26
Manual	2,209	61.46	61.48	3,088	67.54	67.57
No father in HH/Other	254	7.07	7.10	236	5.16	5.17
*Missing*	*284*	*7.32*		*250*	*5.18*	
**Maternal age at birth (mean**,** SD)**	3,609	25.94 (5.32)	25.95 (5.54)	4,568	27.49 (5.67)	27.48 (5.89)
*Missing*	*269*	*6.94*		*258*	*5.27*	
**Previous live births (mean**,** SD)**	3,608	1.09 (1.29)	1.09 (1.32)	4572	1.26 (1.53)	1.26 (1.59)
*Missing*	*270*	*6.96*		*254*	*5.18*	

Note:

^1^ 167 (4.92% of) women in BCS70 and 168 (4.08%) in NCDS were born before 37 weeks of gestation (preterm)

The imputation model included birthweight z-score rather than birthweight (kg) and gestational age (weeks)

Table 2 illustrates the associations between maternal smoking during pregnancy, birthweight z-scores and breastfeeding duration, as well as their relationships with the timing of menopause in the pooled and study specific samples. Maternal cigarette smoking during pregnancy was associated with lower birthweight z-score [β: -0.29; 95% CI -0.34, -0.24] and lower likelihood for longer breastfeeding [RRR_< 1month_: 0.90; 95% CI 0.79, 1.02; RRR_≥ 1 month_: 0.66; 95% CI 0.59, 0.73], relative to no breastfeeding. The likelihood for being breastfed for longer, compared to not being breastfed, increased with an increase in birthweight z-score. One standard deviation higher birthweight z-score corresponded to 5% higher odds for being breastfed for less than one month (RRR_< 1month_: 1.05; 95% CI 0.98, 1.12;) and 6% increased odds for being breastfed for 1 month or longer (RRR_≥ 1 month_: 1.06; 95% CI 1.00, 1.12). An increase in the z-score for birthweight was associated with lower hazard for earlier age at natural menopause (HR: 0.96, 95% CI 0.91, 1.01). Women who were breastfed for 1 month or longer had a lower hazard of earlier menopause (HR: 0.84, 95% CI 0.74, 0.96) compared to women who never received breastmilk. The patterns of these associations were similar in the study specific analyses, though the evidence for an association between breastfeeding and age at menopause in NCDS was weaker (Table 2).

**Table 2:** Associations between maternal smoking during pregnancy, birthweight z-scores, breastfeeding duration, and time to natural menopause in daughters (imputed sample)

Pooled (*n* = 8,700, followed-up to age 50 years)	Birthweight z-score	Breastfed < 1 month reference: (never)	Breastfed 1 + months reference: (never)	Daughter’s experience of natural menopause reference: no (incl. pre-, peri-, surgical menopause, HT)
	β (95% CI)	RRR (95% CI)	RRR (95% CI)	HR (95% CI)
**Maternal smoking in pregnancy** (reference: no smoking)	-0.29 (-0.34, -0.24) ^1^	0.90 (0.79, 1.02)	0.66 (0.59, 0.73) ^1^	1.13 (1.01, 1.26) ^1^
**Birthweight z-score**		1.05 (0.98, 1.12)	1.06 (1.00, 1.12) ^2^	0.96 (0.91, 1.01) ^2^
**Breastfed < 1 month** (reference: never)				0.99 (0.86, 1.15) ^3^
**Breastfed 1month +** (reference: never)				0.84 (0.74, 0.96) ^3^
**BCS70** (*n* = 3,878, followed-up to age 46 years)	**Birthweight z-score**	**Breastfed < 1 month** reference: (never)	**Breastfed 1 + months** reference: (never)	**Daughter’s experience of natural menopause** reference: no (incl. pre-, peri-, surgical menopause, HT)
	β (95% CI)	RRR (95% CI)	RRR (95% CI)	HR (95% CI)
**Maternal smoking in pregnancy** (reference: no smoking)	-0.32 (-0.40, -0.24) ^1^	0.82 (0.66, 1.01)	0.62 (0.51, 0.75) ^1^	1.21 (0.97, 1.51) ^1^
**Birthweight z-score**		1.03 (0.93, 1.14)	1.07 (0.98, 1.17) ^2^	1.00 (0.90, 1.10) ^2^
**Breastfed < 1 month** (reference: never)				0.91 (0.66, 1.27) ^3^
**Breastfed 1month +** (reference: never)				0.47 (0.33, 0.68) ^3^
**NCDS** (*n* = 4,822, followed-up to age 50 years)	**Birthweight z-score**	**Breastfed < 1 month** reference: (never)	**Breastfed 1 + months** reference: (never)	**Daughter’s experience of natural menopause** reference: no (incl. pre-, peri-, surgical menopause, HT)
	β (95% CI)	RRR (95% CI)	RRR (95% CI)	HR (95% CI)
**Maternal smoking in pregnancy** (reference: no smoking)	-0.27 (-0.34, -0.20) ^1^	0.99 (0.84, 1.17)	0.71 (0.61, 0.83) ^1^	1.10 (0.97, 1.24) ^1^
**Birthweight z-score**		1.06 (0.98, 1.16)	1.06 (0.98, 1.13) ^2^	0.95 (0.89, 1.00) ^2^
**Breastfed < 1 month** (reference: never)				1.06 (0.90, 1.25) ^3^
**Breastfed 1month +** (reference: never)				0.96 (0.83, 1.11) ^3^

Note:

Model adjusted for:

^1^ maternal education, social class and maternal age at birth, previous live births (and cohort in pooled sample)

^2^ maternal education, social class and maternal age at birth, previous live births, maternal smoking in pregnancy (and cohort in pooled sample)

^3^ maternal education, social class and maternal age at birth, previous live births, maternal smoking in pregnancy, birthweight z-score (and cohort in pooled sample)

Results from complete cases are presented in Supplementary material (Table S3)

The estimated total causal effects, direct and indirect effects, and the corresponding bias-corrected bootstrap confidence intervals, in the pooled and study specific samples, are shown in Table 3. Maternal smoking during pregnancy, compared to non-smoking, increased the hazard of daughter’s menopause by 13% (HR_TE_=1.13, 95% CI 1.02, 1.24). Birthweight z-score alone mediated an estimated 5.8% of the TE (HR_NIE_= 1.01, 95% CI 0.99, 1.04), and jointly birthweight z-score and breastfeeding mediated an estimated 14.4% (HR_NIE_=1.02, 95% CI 0.99, 1.05). Independent of birthweight-z-score and breastfeeding duration, maternal smoking in pregnancy increased the hazard of daughter’s menopause by 12% and 11%, respectively (birthweight z-score HR_NDE_ 1.12, 95% CI 1.02, 1.24; birthweight z-score and breastfeeding duration HR_NDE_=1.11, 95% CI 1.01, 1.23).

The pattern of results was similar across the cohorts, but all effects were stronger in BCS70 than in NCDS (Table 3). In BCS70, maternal smoking during pregnancy increased the hazard of daughter’s menopause by 21% (HR_TE_=1.21, 95% CI 0.97, 1.52). Birthweight z-score alone mediated an estimated 4.1% of this association (HR_NIE_= 1.01, 95% CI 0.94, 1.08), while jointly birthweight z-score and breastfeeding mediated an estimated 22.1% (HR_NIE_=1.04, 95% CI 0.96, 1.13). Independent of birthweight-z-score and breastfeeding, maternal smoking in pregnancy increased the hazard of daughter’s menopause by 20% and 16%, respectively (birthweight z-score HR_NDE_ 1.20, 95% CI 0.94, 1.58; birthweight z-score and breastfeeding HR_NDE_=1.16, 95% CI 0.90, 1.52). In NCDS, prenatal cigarette smoking increased the hazard of menopause by only 10% (HR_TE_=1.10, 95% CI 0.99, 1.22) and birthweight z-score mediated a smaller percentage of the association estimated at 1.7% (HR_NIE_=1.00 95% CI 0.98, 1.04). The joint mediation with breastfeeding was also less than for BCS70 estimated at 6.4% (HR_NIE_=1.01, 95% CI 0.98, 1.05). As in BCS70, independent of birthweight z-score and breastfeeding, prenatal cigarette smoking increased the hazard of menopause by 10% and 9%, respectively (birthweight z-score HR_NDE_ 1.10, 95% CI 0.99, 1.22; birthweight z-score and breastfeeding HR_NDE_=1.09, 95% CI 0.99, 1.23).

**Table 3:** Mediation of the effect of maternal smoking in pregnancy on time to natural menopause in daughters by birthweight z-scores and breastfeeding duration (imputed sample)

Pooled sample (*n* = 8,700, followed-up to age 50 years)	Natural menopause		
	HR	BCB 95% CI	
Mediation by birthweight z-score			
Total	1.13	1.02	1.24
Indirect (acting through the mediators)	1.01	0.99	1.04
Direct (unexplained by these mediators)	1.12	1.02	1.24
proportion mediated (%)	**5.83**		
Mediation by birthweight z-score + breastfeeding duration			
Total	1.13	1.02	1.24
Indirect	1.02	0.99	1.05
Direct	1.11	1.01	1.23
proportion mediated (%)	**14.39**		
**BCS70 (** ***n*** ** = 3,878, followed-up to age 46 years)**	Natural menopause		
	HR	BCB 95% CI	
Mediation by birthweight z-score			
Total	1.21	0.97	1.52
Indirect	1.01	0.94	1.08
Direct	1.20	0.94	1.58
proportion mediated (%)	**4.05**		
Mediation by birthweight z-score + breastfeeding duration			
Total	1.21	0.97	1.52
Indirect	1.04	0.96	1.13
Direct	1.16	0.90	1.52
proportion mediated (%)	**22.05**		
**NCDS (** ***n*** ** = 4,822, follow-up to age 50 years)**	Natural menopause		
	HR	BCB 95% CI	
Mediation by birthweight z-score			
Total	1.10	0.99	1.22
Indirect	1.00	0.98	1.04
Direct	1.10	0.99	1.22
proportion mediated (%)	**1.73**		
Mediation by birthweight z-score + breastfeeding duration			
Total	1.10	0.99	1.22
Indirect	1.01	0.98	1.05
Direct	1.09	0.99	1.23
proportion mediated (%)	**6.42**		

Note:

Bias-corrected bootstrap 95% CIs; bootstrapping based on 200 replications

The proportion mediated was calculated using the formula: {HRNDE (HRNIE − 1)/(HRNDE * HRNIE − 1)}*100

Models adjusted for maternal education, social class and maternal age at birth, previous live births (and cohort in pooled sample)

Results from complete cases are presented in Supplementary material (Table S4)

### Secondary analysis

We repeated the analysis by restricting the follow-up time in the pooled sample to the follow-up period in the younger BCS70 cohort (up to age 46 years) and observed small increases in the proportions mediated by birthweight z-score and jointly with breastfeeding, but a similar pattern to the results with varying follow-up periods. Birthweight z-score alone mediated an estimated 12.9% (HR_NIE_= 1.01, 95% CI 0.98, 1.05) and jointly with breastfeeding mediated an estimated 20.5% (HR_NIE_=1.02, 95% CI 0.99, 1.06) (Table S6, Supplementary material).

## Discussion

Our results provide some support for a hypothesised pathway whereby maternal smoking during pregnancy influences menopausal age in daughters, partially through fetal growth and breastfeeding duration. We have shown that prenatal exposure to cigarette smoke was related to increasing hazard of earlier menopause and lower birthweight z-score and lack of breastfeeding accounted for an estimated 14% of the increased hazard of earlier menopause. After accounting for the effect of birthweight z-score and breastfeeding, there remained evidence of a direct effect of maternal smoking during pregnancy on the timing of daughters’ menopause. This leaves room for unmeasured mediators or other interlinked mediating pathways including a direct influence of maternal smoking during pregnancy. The pattern of results was similar across the cohorts though some differences in the effect sizes were noticeable, possibly due to different confounding structures. These results also followed a similar pattern in the secondary analysis, where the follow-up period was restricted to 46 years of age (the follow-up time in the younger cohort).

To our knowledge, the role of intermediate factors in the effect of prenatal exposure to cigarette smoking on time to natural menopause in female offspring has not been quantified previously. Consistent with previous research, we illustrated that fetal growth and breastfeeding duration are both influenced by maternal smoking in pregnancy and that breastfeeding duration is influenced by fetal growth [21, 41]. We also showed that greater birthweight z-score and breastfeeding duration are associated with lower hazards for earlier menopause [12, 14]. These relationships provided the foundation for the hypothesised mediation in this study.

There are several possible pathways through which fetal growth may influence the time to menopause. Restricted fetal growth has been associated with adverse environment in fetal life and suboptimal fetal development, which in turn may increase the rate of follicle atresia during fetal life and reduce the ovarian follicle reserve at birth [42, 43]. Restricted fetal growth may also contribute to permanent changes in physiology and metabolism which, in turn, may increase the risk of several diseases in later life and contribute to follicle loss after birth [44]. Low birthweight has also been related to suboptimal breastfeeding outcomes for which aspects of childbirth hospital care, infant and maternal factors may play a role [22, 24]. Breast milk activates several metabolic processes influencing microanatomy development, growth, metabolism, gut microbiological colonisation and maturation, immunological and brain systems development [45]. Disease protection, optimal growth and improved cognitive development may in turn constitute some of the possible pathways through which breastfeeding may influence the timing of the menopause.

### Implications

Our analysis suggests that a small part of the harmful effect of cigarette smoking during pregnancy on the reproductive longevity of the female offspring may be offset through maternal focused interventions to improve birth and breastfeeding outcomes. However, efforts to discourage smoking during pregnancy may play an even stronger role, noting the possibility of a direct effect of maternal smoking in utero on daughter’s timing of menopause. There has been a marked increase in the rates of breastfeeding in the UK in recent years, however, mothers who smoke are less likely to breastfeed [46, 47]. According to qualitative studies, mothers who smoke base their intentions to breastfeed on how they perceive the health risks that smoking poses to their newborn. Many smoking mothers think that formula is better for their newborn than their milk, which contains nicotine and other toxins [48, 49]. Public health advice provided by the NHS, CDC and others already recommends that mothers breastfeed even if they cannot stop smoking; [50, 51] further efforts to promote the benefits of breastfeeding among smokers could be beneficial. Women who smoke are also more likely to have low milk supply which limits their ability to breastfeed [47]. Women facing these difficulties should receive further support [22]. 

As breastfeeding and birth weight only mediated a small proportion of the effect of maternal smoking on daughters’ reproductive longevity, there may be other pathways involved. Therefore, more research is needed on the factors that mediate this effect, especially those that are modifiable. As mentioned, it also may be that cigarette smoking during pregnancy has a direct impact (i.e. not via any previously hypothesised mediators) on the reproductive health of the female offspring. While the epidemiological evidence of this direct effect is inconclusive, one possible explanation is the adjustment for variables that may mediate the effect of smoking during pregnancy on the age of menopause in the female offspring in previous studies [5, 6, 8]. We therefore recommend further research takes into account the temporal sequence of factors adjusted.

### Strengths and limitations

Our study does have limitations. As with any longitudinal study, both cohorts have been affected by attrition. Fortunately, the retention rates over the decades following the study members have been strong [52]. The size of our analytical sample was also affected by insufficient information to derive menopause status (sometimes due to survey error) and intentional exclusions based on the definition of our target population. We approached the missing data problem with MI. Beyond being of substantive interest in this study, including birth and early life characteristics such as social class at birth, maternal age at birth, and breastfeeding in our imputation models can help reduce bias due to missing data and restore sample representativeness [52, 53]. Despite our efforts, bias due to selective attrition cannot be ruled out.

We define as ‘smokers’ women who smoked at any time during the pregnancy, and we do not have information about when these women stopped smoking or whether they smoked during lactation. However, we included women who smoked at any time because clinical research has illustrated irreversible effect of prenatal cigarette smoke exposure on germ and somatic cells in female gonads as early as the first trimester [3]. We did not have information on ‘exclusive’ breastfeeding; our information on breastfeeding relates to any breastfeeding. This means that the breastfed groups in our analysis may include those who have received formula alongside breast milk. Our data also do not allow us to explore the effects of specific breastfeeding lengths longer than 1 month. Nonetheless, to better understand the impact of breastfeeding duration, we categorized women who were breastfed for periods shorter and longer than one month separately. Smoking during pregnancy and infant feeding behaviours were self-reported by the cohort women’s mothers, and there is potential for misclassification which (if differential) may distort the exposure-outcome associations. However, we consider the potential for misclassification due to socially desirable response small, as both smoking and formula feeding were considered normative in the late 50s and early 70s [54]. Measurement error in the exposure and mediator variables can contribute to bias in the causal mediation analysis and potentially underestimate the indirect effect, therefore, the proportion mediated [55, 56]. Other limitations include the retrospective collection of information on breastfeeding and menopause and potential for recall bias although recall was not over a long period.

Like other counterfactual-based approaches to mediation, the IOW method assumes no unmeasured confounding of the exposure-outcome effect, the mediator-outcome effect, and the exposure-mediator effect. Further, the IOW method assumes that there are no confounders of the mediator-outcome effect that are affected by the exposure [36, 37]. Despite our best attempts to account for important determinants of maternal smoking, fetal growth, breastfeeding, and menopausal age, unmeasured confounding cannot be ruled out. To minimize potential violation of the no intermediate confounding assumption, we investigated the role of potential mediators operating in period of life which is close to the exposure than to the outcome. The closer in time the mediator is measured as compared with the exposure, the less likely this assumption is to be violated. Another limitation of the IOW method is that the variances of estimates can be wider than those of traditional parametric mediation methods making it more difficult to detect small indirect effects. Coefficients and effect sizes in causal mediation analysis are often small due to attrition, measurement error, and use of multiple mediators. These limitations are best handled by increasing the statistical power of the analysis, as well as optimising the temporal interval between the exposure and mediators, and using bootstrap confidence intervals [56], as in our analysis.

The strengths of our analysis are the use of prospective birth cohort studies following people throughout life which offer advantages for studying intergenerational transmission of disadvantage in health. NCDS and BCS70 provide a unique opportunity to study the effect of maternal smoking during pregnancy on the time to menopause in daughters which younger cohort studies cannot yet offer. The comparable study designs, measures, and follow-up periods, allow us to combine the data from the two cohorts and perform pooled analysis on imputed data with increased statistical power. The rates of smoking during pregnancy in the studied cohorts are considerably higher than those in recent years in the UK (less than 10% of mothers smoked during pregnancy in 2021/22) [50]. The rates of breastfeeding were also low compared to recent figures (about 72% of babies had a first feed of breast milk in 2022/23); [57, 58] even though the data are not exactly comparable due to differences in breastfeeding definitions. This could be considered a further methodological advantage for this analysis as it may help in detecting an effect; but it can also be a potential issue for generalisability to contemporary cohorts. Although it has limitations, the IOW method offers the advantage to estimate causally interpretable effects in the context of multiple mediators irrespective of their measurement scale, in a time-to-event setting, and with imputed datasets, and further, in the presence of exposure-mediator interactions. Weighting treats the exposure and mediators as independent by deactivating indirect pathways of the mediators. IOW is agnostic with regards to effects of interactions and thus is valid regardless of interactions between any set of confounders, exposure, or mediators on the outcome, without the need to specify them [36, 37]. 

## Conclusion

Our research found that birthweight-for-gestational-age-z-score and breastfeeding together mediated an estimated 14% of the effect of prenatal exposure to maternal cigarette smoking on the timing of menopause in female offspring. This suggests that early life factors could potentially counteract the harmful effects of maternal smoking during pregnancy, to the extent that they mediate the risk of earlier menopause. Alongside interventions to stop smoking, providing education and support for breastfeeding in the early postnatal period could contribute to women’s reproductive longevity. However, since breastfeeding and birth weight mediated just 14% of the effect of prenatal maternal smoking and daughters’ reproductive longevity, there is a need for further research on the factors that mediate this effect.

## Electronic supplementary material

Below is the link to the electronic supplementary material.

Additional file 1: Title of data: Supplementary material: Mediation of the effect of prenatal maternal smoking on time to natural menopause in daughters by birthweight-for-gestational-age z-score and breastfeeding duration: Analysis of two UK birth cohorts born in 1958 and 1970. Description of data: Figure S1. Participation and attrition in the 1970 British Cohort Study (A) and 1958 National Child Development Study (B); Table S1. Classification of women into menopause status in the 1970 British Cohort Study (A) and 1958 National Child Development Study (B); Table S2. Missing data in the dates of menopause, surgery, or HT initiation in the 1970 British Cohort Study (A) and 1958 National Child Development Study (B); Table S3. Associations between maternal smoking during pregnancy, birthweight z-scores, breastfeeding duration, and time to natural menopause in daughters (complete case sample); Table S4. Mediation of the effect of maternal smoking in pregnancy on time to natural menopause in daughters by birthweight z-scores and breastfeeding (complete case sample); Table S5. Crude associations between maternal smoking during pregnancy, birthweight z-scores, breastfeeding duration, time to natural menopause in daughters, and potential confounders (imputed samples); Table S6. Mediation of the effect of maternal smoking in pregnancy on time to natural menopause in daughters by birthweight z-scores and breastfeeding (imputed sample)

## Data Availability

The study uses aggregated pseudonymised data publicly available at the UK Data Service (https://ukdataservice.ac.uk/).

## Abbreviations

BCS70: 1970 British Cohort Study
NCDS: 1958 National Child Development Study
FMP: Final menstrual period
HT: Hormonal therapy
SD: Standard deviation
CI: Confidence interval
BCB CI: Bias corrected bootstrap confidence interval
RRR: Relative Risk Ratio
HR: Hazard Ratio
TE: Total effect
NDE: Natural direct effect
NIE: Natural indirect effect
MI: Multiple imputation
