# Supplementary material for: Mediation of the effect of prenatal maternal smoking on time to natural menopause in daughters by birthweight-for-gestational-age z-score and breastfeeding duration: analysis of two UK birth cohorts born in 1958 and 1970
Source: BMC Womens Health. 2025 Jan 23;25:32. doi: 10.1186/s12905-025-03556-y (PMC11756059; doi:10.1186/s12905-025-03556-y)
Supplement: Supplementary file 1 — Additional file 1: Title of data: Supplementary material: Mediation of the effect of prenatal maternal smoking on time to natural menopause in daughters by birthweight-for-gestational-age z-score and breastfeeding duration: Analysis of two UK birth cohorts born in 1958 and 1970. Description of data: Figure S1. Participation and attrition in the 1970 British Cohort Study (A) and 1958 National Child Development Study (B); Table S1. Classification of women into menopause status in the 1970 British Cohort Study (A) and 1958 National Child Development Study (B); Table S2. Missing data in the dates of menopause, surgery, or HT initiation in the 1970 British Cohort Study (A) and 1958 National Child Development Study (B); Table S3. Associations between maternal smoking during pregnancy, birthweight z-scores, breastfeeding duration, and time to natural menopause in daughters (complete case sample); Table S4. Mediation of the effect of maternal smoking in pregnancy on time to natural menopause in daughters by birthweight z-scores and breastfeeding (complete case sample); Table S5. Crude associations between maternal smoking during pregnancy, birthweight z-scores, breastfeeding duration, time to natural menopause in daughters, and potential confounders (imputed samples); Table S6. Mediation of the effect of maternal smoking in pregnancy on time to natural menopause in daughters by birthweight z-scores and breastfeeding (imputed sample) [file 12905_2025_3556_MOESM1_ESM.docx]

**Supplementary material: Mediation of the effect of prenatal maternal smoking on time to natural menopause in daughters by birthweight-for-gestational-age z-score and breastfeeding duration: analysis of two uk birth cohorts born in 1958 and 1970**

**A)**


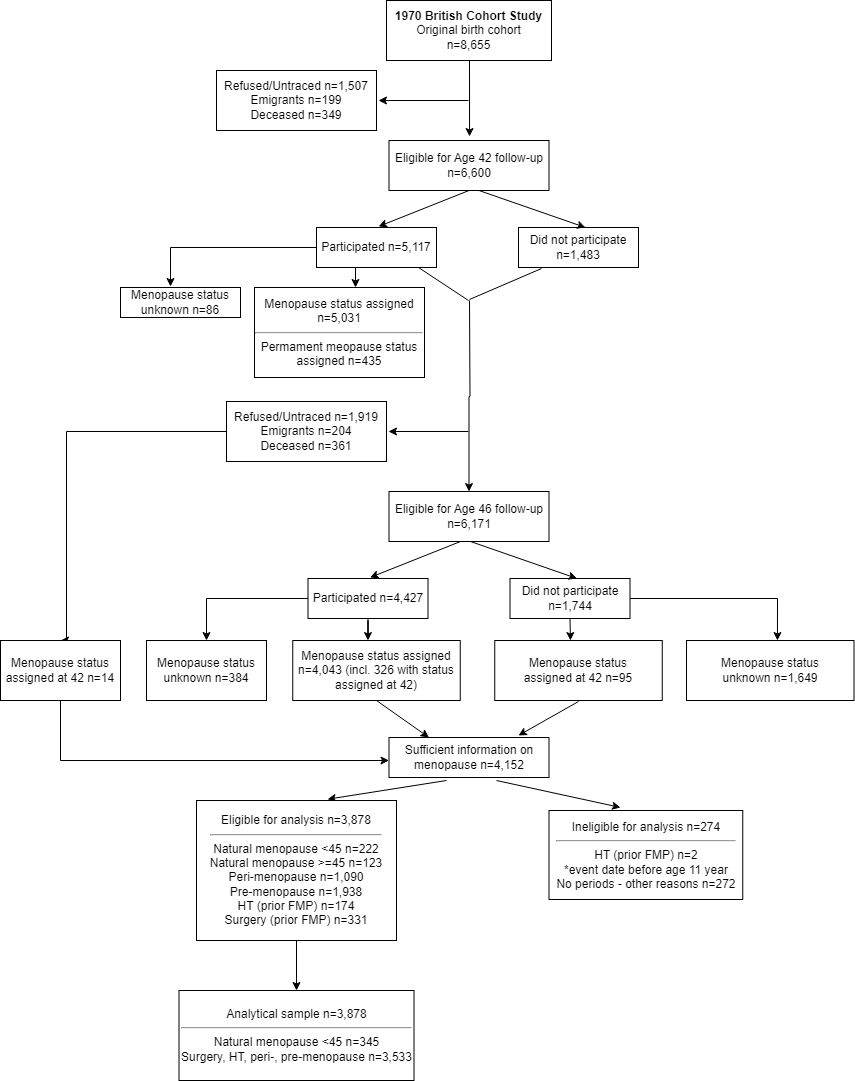


**B)**


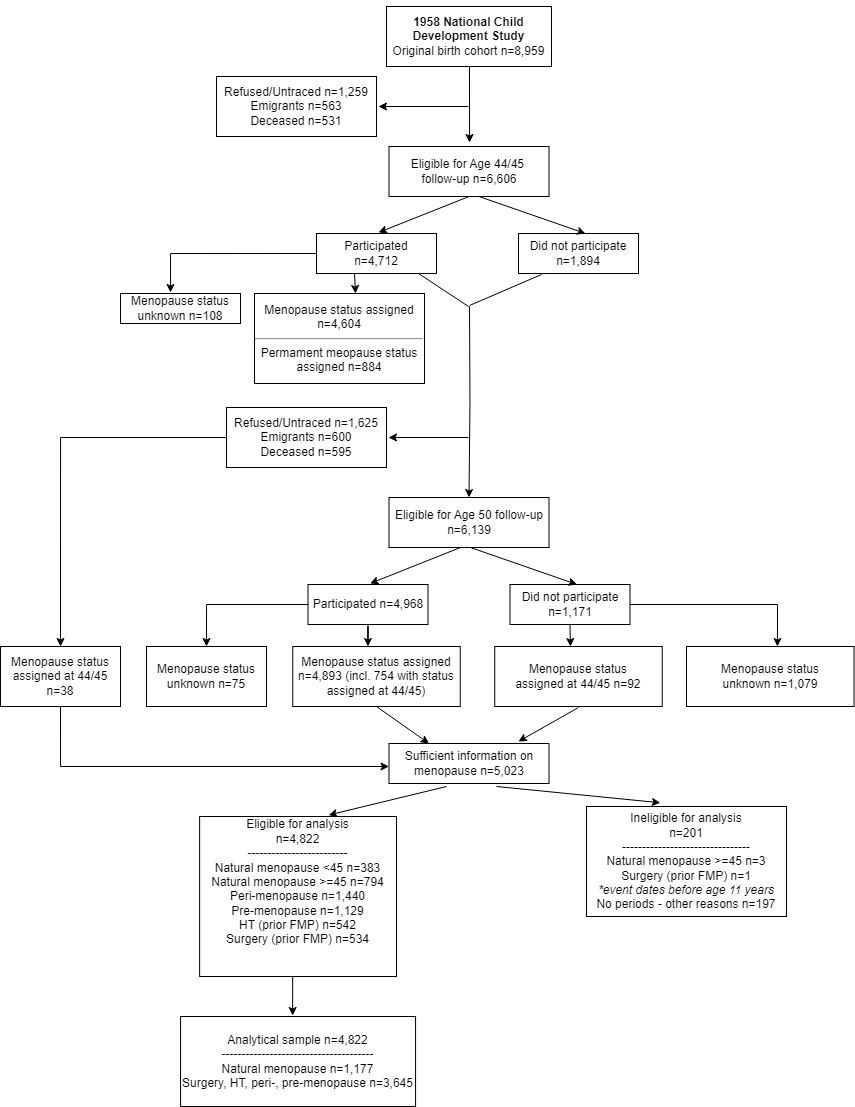
**Figure S1** Participation and attrition in the 1970 British Cohort Study (A) and 1958 National Child Development Study (B)

**Table S1.** Classification of women into menopause status in the 1970 British Cohort Study (A) and 1958 National Child Development Study (B)

A)

| **1970 BCS cohort** | | | | |
| --- | --- | --- | --- | --- |
| **Menopause status** | **Age 42** | | **Age 46** | |
|  | n | % | n | % |
| Menopause <40 years  (premature menopause) | 44 | 0.9 | 53 | 1.2 |
| Menopause 40-44 years (early menopause) | 86 | 1.7 | 169 | 3.7 |
| Menopause 45 or more years | n.a. |  | 123 | 2.7 |
| Peri-menopause | 812 | 15.9 | 1,090 | 24.0 |
| Pre-menopause | 3,390 | 66.2 | 1,938 | 42.7 |
| Hormone therapy (before FMP) | 72 | 1.4 | 176 | 3.9 |
| Hysterectomy/Bilateral oophorectomy  (before FMP) | 182 | 3.6 | 331 | 7.3 |
| No periods (other reasons) | 414 | 8.1 | 241 | 5.3 |
| Never had a period | 31 | 0.6 | 31 | 0.7 |
| Insufficient information | 86 | 1.7 | [384 ^[1]^](file:///C:\Users\sejjdnp\AppData\Local\Microsoft\Windows\INetCache\Content.MSO\7CC66F39.xlsx#RANGE!_ftn1) | 8.5 |
| (Age 42 participants) Not in age 46 survey | |  | 878 |  |
| Total | 5,117 | 100.0 | 4,536 | 100.0 |
|  |  |  |  |  |
| *Notes:  If a woman had gone through natural menopause or a surgery (hysterectomy/bilateral oophorectomy) or had initiated HT prior to the FMP, their menopausal status remained unchanged for the subsequent survey (i.e. figures at age 46 include women with permanent menopause status at age 42).* | | | | |
| *Overall number of women (and denominator) excludes cases non-participating in subsequent sweep (i.e. 878 women who took part at age 42 but not age 46).* | | | | |
| *Women whose periods stopped for other obvious reasons (e.g. pregnancy, contraceptives, chemotherapy or radiotherapy) were excluded from this analysis.* | | | | |
| *Analytical sample further excludes 2 women (on HT prior FMP) with reported event date before age 11 years .* | | | | |
| [*[1] Due to an error, women at age 46 in the 1970 cohort who at age 42 reported no periods in the past 12 months for reasons different from natural menopause, including pregnancy and contraceptive use, were not asked whether they had periods in the past 12 months, resulting in insufficient information to determine their menopause status at age 46 survey.*](file:///C:\Users\sejjdnp\AppData\Local\Microsoft\Windows\INetCache\Content.MSO\7CC66F39.xlsx#RANGE!_ftnref1) | | | | |

B)

| **1958 NCDS cohort** | | | | |
| --- | --- | --- | --- | --- |
| **Menopause status** | **Age 44/45** | | **Age 50** | |
|  | n | % | n | % |
| Menopause <40 years  (premature menopause) | 131 | 2.8 | 176 | 3.5 |
| Menopause 40-44 years (early menopause) | 106 | 2.2 | 207 | 4.1 |
| Menopause 45 or more years | 23 | 0.5 | 797 | 15.6 |
| Peri-menopause | 841 | 17.8 | 1,440 | 28.2 |
| Pre-menopause | 2,833 | 60.1 | 1,129 | 22.1 |
| Hormone therapy (before FMP) | 268 | 5.7 | 542 | 10.6 |
| Hysterectomy/Bilateral oophorectomy  (before FMP) | 294 | 6.2 | 535 | 10.5 |
| No periods (other reasons) | 108 | 2.3 | 197 | 3.9 |
| Never had a period | - |  | - |  |
| Insufficient information | 108 | 2.3 | 75 | 1.5 |
| (Age 44 participants) Not in age 50 survey |  |  | 411 |  |
| Total | 4,712 | 100.0 | 5,098 | 100.0 |
|  |  |  |  |  |
| *Notes:  If a woman had gone through natural menopause or a surgery (hysterectomy/bilateral oophorectomy) or had initiated HT prior to the FMP, their menopausal status remained unchanged for the subsequent survey (i.e. figures at age 50 include women with permanent menopause status at age 44/45).* | | | | |
| *Overall number of women (and denominator) excludes cases non-participating in subsequent sweep (i.e. 411 women who took part at age 44/45 but not age 50).* | | | | |
| *Women whose periods stopped for other obvious reasons (e.g. pregnancy, contraceptives, chemotherapy or radiotherapy) were excluded from this analysis.* | | | | |
| *Analytical sample further excludes 4 women with reported event date before age 11 years (3 with natural menopause >=45 years and 1 with surgical menopause).* | | | | |
| *Due to an error, women in the 1958 cohort who reported natural menopause in the age 44/45 survey were not asked about their age at their FMP; they were asked about this subsequently at the age 50 follow-up.* | | | | |

**Table S2.** Missing data in the dates of menopause, surgery, or HT initiation in the 1970 British Cohort Study (A) and 1958 National Child Development Study (B)

A)

| **1970 BCS cohort** | | | | | | |
| --- | --- | --- | --- | --- | --- | --- |
| **Menopause status**  **Missing data** | **Age 42** | | | **Age 46** | | |
|  | n | n missing | %  missing | n | n  missing | %  missing |
| Menopause  Missing month of FMP  Missing month and year of FMP | 110 | 32  55 | 29.1  50.0 | 345 | 40  71 | 11.6  20.1 |
| Hormone therapy (before FMP)  Missing month of HT initiation  Missing month and year of HT initiation | 72 | 20  14 | 27.8  19.4 | 176 | 24  16 | 13.6  9.1 |
| Hysterectomy/Bilateral oophorectomy (before FMP)  Missing month of surgery  Missing month and year of surgery | 182 | 13  7 | 7.1  3.8 | 331 | 14  9 | 4.2  2.7 |

*Notes: N at age 46 includes n at age 42 (i.e. n at 46 is cumulative).*

B)

| **1958 NCDS cohort** | | | | | | |
| --- | --- | --- | --- | --- | --- | --- |
| **Menopause status**  **Missing data** | **Age 44/45** | | | **Age 50** | | |
|  | n | n missing | %  missing | n | n  missing | %  missing |
| Menopause  Missing month of FMP  Missing month and year of FMP | 260 | 6  38 | 2.3  14.6 | 1,180 | 60  52 | 5.1  4.4 |
| Hormone therapy (before FMP)  Missing month of HT initiation  Missing month and year of HT initiation | 268 | 106  10 | 39.6  3.7 | 542 | 119  16 | 22.0  3.0 |
| Hysterectomy/Bilateral oophorectomy (before FMP)  Missing month of surgery  Missing month and year of surgery | 294 | 66  23 | 22.4  7.8 | 535 | 75  28 | 14.0  5.2 |

*Notes: N at age 50 includes n at age 44/45 (i.e. n at 50 is cumulative).*

**Table S3.** Associations between maternal smoking during pregnancy, birthweight z-scores, breastfeeding duration, and time to natural menopause in daughters (complete case sample)

| **Pooled**  (n=6,354, followed-up to age 50 years) | **Birthweight z-score** | **Breastfed <1 month** reference: (never) | **Breastfed 1+months** reference: (never) | **Daughter’s experience of natural menopause**  reference: no (incl. pre-, peri-, surgical menopause, HT) |
| --- | --- | --- | --- | --- |
|  | **β (95% CI)** | **RRR (95% CI)** | **RRR (95% CI)** | **HR (95% CI)** |
| **Maternal smoking in pregnancy** (reference: no smoking) | -0.28 (-0.33, -0.22) ^1^ | 0.92 (0.80, 1.05) | 0.66 (0.59, 0.75) ^1^ | 1.19 (1.06, 1.35) ^1^ |
| **Birthweight z-score** |  | 1.06 (0.99, 1.12) | 1.05 (1.00, 1.11) ^2^ | 0.95 (0.90, 1.00) ^2^ |
| **Breastfed <1 month** (reference: never) |  |  |  | 0.97 (0.83, 1.14) ^3^ |
| **Breastfed 1month +** (reference: never) |  |  |  | 0.82 (0.71, 0.94) ^3^ |
| **BCS70**  (n=2,826, followed-up to age 46 years) | **Birthweight z-score** | **Breastfed <1 month** reference: (never) | **Breastfed 1+months** reference: (never) | **Daughter’s experience of natural menopause**  reference: no (incl. pre-, peri-, surgical menopause, HT) |
|  | β (95% CI) | RRR (95% CI) | RRR (95% CI) | HR (95% CI) |
| **Maternal smoking in pregnancy** (reference: no smoking) | -0.29 (-0.38, -0.21) ^1^ | 0.85 (0.68, 1.05) | 0.64 (0.52, 0.77) ^1^ | 1.34 (1.04, 1.73) ^1^ |
| **Birthweight z-score** |  | 1.03 (0.93, 1.14) | 1.06 (0.97, 1.16) ^2^ | 1.00 (0.89, 1.12) ^2^ |
| **Breastfed <1 month** (reference: never) |  |  |  | 0.90 (0.64, 1.27) ^3^ |
| **Breastfed 1month +** (reference: never) |  |  |  | 0.41 (0.27, 0.62) ^3^ |
| **NCDS**  (n=3,528, followed-up to age 50 years) | **Birthweight z-score** | **Breastfed <1 month** reference: (never) | **Breastfed 1+months** reference: (never) | **Daughter’s experience of natural menopause**  reference: no (incl. pre-, peri-, surgical menopause, HT) |
|  | β (95% CI) | RRR (95% CI) | RRR (95% CI) | HR (95% CI) |
| **Maternal smoking in pregnancy** (reference: no smoking) | -0.26 (-0.33, -0.19) ^1^ | 1.01 (0.84, 1.21) | 0.71 (0.61, 0.84) ^1^ | 1.15 (1.01, 1.32) ^1^ |
| **Birthweight z-score** |  | 1.07 (0.99, 1.17) | 1.05 (0.98, 1.13) ^2^ | 0.94 (0.88, 1.00) ^2^ |
| **Breastfed <1 month** (reference: never) |  |  |  | 1.04 (0.87, 1.24) ^3^ |
| **Breastfed 1month +** (reference: never) |  |  |  | 0.93 (0.79, 1.09) ^3^ |

Note:

Model adjusted for:

^1^ maternal education, social class and maternal age at birth, previous live births (and cohort in pooled sample)

^2^ maternal education, social class and maternal age at birth, previous live births, maternal smoking in pregnancy (and cohort in pooled sample)

^3^ maternal education, social class and maternal age at birth, previous live births, maternal smoking in pregnancy, birthweight z-score (and cohort in pooled sample).

**Table S4.** Mediation of the effect of maternal smoking in pregnancy on time to natural menopause in daughters by birthweight z-scores and breastfeeding (complete case sample)

| Pooled sample (n=6,354, followed-up to age 50 years) | Natural menopause | | |
| --- | --- | --- | --- |
|  | HR | BCB 95% CI | |
| Mediation by birthweight z-score | | | |
| Total | 1.19 | 1.06 | 1.34 |
| Indirect (acting through the mediators) | 1.01 | 0.98 | 1.05 |
| Direct (unexplained by these mediators) | 1.18 | 1.03 | 1.33 |
| proportion mediated (%) | **8.42** |  |  |
| Mediation by birthweight z-score + breastfeeding duration | | | |
| Total | 1.19 | 1.06 | 1.34 |
| Indirect | 1.03 | 0.99 | 1.07 |
| Direct | 1.16 | 1.02 | 1.31 |
| proportion mediated (%) | **17.37** |  |  |
| BCS70 (n=2,826 followed-up to age 46 years) | Natural menopause | | |
|  | HR | BCB 95% CI | |
| Mediation by birthweight z-score | | | |
| Total | 1.34 | 1.03 | 1.74 |
| Indirect | 1.03 | 0.95 | 1.12 |
| Direct | 1.29 | 0.99 | 1.70 |
| proportion mediated (%) | **13.33** |  |  |
| Mediation by birthweight z-score + breastfeeding duration | | | |
| Total | 1.34 | 1.03 | 1.74 |
| Indirect | 1.08 | 0.99 | 1.18 |
| Direct | 1.24 | 0.94 | 1.63 |
| proportion mediated (%) | **30.20** |  |  |
| NCDS (n=3,528**,** follow-up to age 50 years) | Natural menopause | | |
|  | HR | BCB 95% CI | |
| Mediation by birthweight z-score | | | |
| Total | 1.15 | 1.00 | 1.32 |
| Indirect | 1.00 | 0.96 | 1.04 |
| Direct | 1.15 | 1.00 | 1.33 |
| proportion mediated (%) | **2.09** |  |  |
| Mediation by birthweight z-score + breastfeeding duration | | | |
| Total | 1.15 | 1.00 | 1.32 |
| Indirect | 1.01 | 0.97 | 1.06 |
| Direct | 1.14 | 0.99 | 1.32 |
| proportion mediated (%) | **7.27** |  |  |

Note:

Bias-corrected bootstrap 95% CIs, bootstrapping based on 10,000 replications.

The proportion mediated was calculated using the formula: {HRNDE (HRNIE− 1)/(HRNDE * HRNIE− 1)}*100.

Models adjusted for maternal education, social class and maternal age at birth, previous live births (and cohort in pooled sample).

**Table S5.** Crude associations between maternal smoking during pregnancy, birthweight z-scores, breastfeeding duration, time to natural menopause in daughters, and potential confounders (imputed samples)

| **BCS70**  (n=3,878, followed-up to age 46 years) | **Maternal smoking in pregnancy** (reference: no smoking) | **Birthweight z-score** | **Breastfed <1 month** reference: (never) | **Breastfed 1+months** reference: (never) | **Daughter’s experience of natural menopause**  reference: no (incl. pre-, peri-, surgical menopause, HT) |
| --- | --- | --- | --- | --- | --- |
|  | OR (95% CI) | β (95% CI) | RRR (95% CI) | RRR (95% CI) | HR (95% CI) |
| Mother in school after minimum school leaving age of 15 years (yes, no) | 0.47 (0.41, 0.55) | 0.10 (0.02, 0.18) | 1.50 (1.21, 1.85) | 3.15 (2.62, 3.79) | 0.67 (0.52, 0.85) |
| Father’s social class at birth - manual (reference: non-manual) | 1.79 (1.54, 2.08) | -0.16 (-0.24, -0.08) | 0.70 (0.56, 0.87) | 0.41 (0.34, 0.49) | 1.47 (1.13, 1.90) |
| Father’s social class at birth - no father figure (reference: non-manual) | 2.59 (1.97, 3.42) | -0.28 (-0.45, -0.12) | 1.11 (0.73, 1.70) | 0.41 (0.28, 0.61) | 1.55 (0.99, 2.42) |
| Maternal age at birth (in years) | 0.97 (0.96, 0.98) | 0.02 (0.01, 0.03) | 0.99 (0.97, 1.00) | 1.02 (1.01, 1.04) | 0.98 (0.96, 1.00) |
| (Number of) previous live births | 1.05 (1.00, 1.11) | 0.09 (0.06, 0.12) | 0.81 (0.74, 0.89) | 0.90 (0.84, 0.97) | 1.04 (0.96, 1.13) |
| **NCDS**  (n=4,822, followed-up to age 50 years) | **Maternal smoking in pregnancy** (reference: no smoking) | **Birthweight z-score** | **Breastfed <1 month** reference: (never) | **Breastfed 1+months** reference: (never) | **Daughter’s experience of natural menopause**  reference: no (incl. pre-, peri-, surgical menopause, HT) |
|  | OR (95% CI) | β (95% CI) | RRR (95% CI) | RRR (95% CI) | HR (95% CI) |
| Mother in school after minimum school leaving age of 15 years (yes, no) | 0.59 (0.51, 0.67) | 0.08 (0.00, 0.16) | 1.16 (0.94, 1.42) | 2.01 (1.70, 2.37) | 0.71 (0.62, 0.82) |
| Father’s social class at birth - manual (reference: non-manual) | 1.60 (1.39, 1.83) | -0.15 (-0.23, -0.08) | 1.12 (0.92, 1.37) | 0.65 (0.55, 0.76) | 1.46 (1.27, 1.68) |
| Father’s social class at birth - no father figure (reference: non-manual) | 2.19 (1.65, 2.89) | -0.30 (-0.48, -0.12) | 0.91 (0.62, 1.35) | 0.38 (0.27, 0.54) | 1.63 (1.24, 2.13) |
| Maternal age at birth (in years) | 1.02 (1.01, 1.03) | 0.02 (0.02, 0.03) | 0.95 (0.94, 0.96) | 0.97 (0.96, 0.98) | 1.00 (0.99, 1.01) |
| (Number of) previous live births | 1.09 (1.05, 1.13) | 0.10 (0.07, 0.12) | 0.86 (0.82, 0.91) | 0.86 (0.81, 0.90) | 1.09 (1.06, 1.13) |

**Table S6.** Mediation of the effect of maternal smoking in pregnancy on time to natural menopause by birthweight z-scores and breastfeeding (imputed sample)

| **Pooled sample (n=8,700, followed-up to age 48 years)** | **Natural menopause** | | |
| --- | --- | --- | --- |
|  | HR | BCB 95% CI | |
| Mediation by birthweight z-score | | | |
| Total | 1.10 | 0.99 | 1.22 |
| Indirect (acting through the mediators) | 1.01 | 0.98 | 1.05 |
| Direct (unexplained by these mediators) | 1.09 | 0.98 | 1.22 |
| proportion mediated (%) | **12.87** |  |  |
| Mediation by birthweight z-score + breastfeeding duration | | | |
| Total | 1.10 | 0.99 | 1.22 |
| Indirect | 1.02 | 0.99 | 1.06 |
| Direct | 1.08 | 0.96 | 1.21 |
| proportion mediated (%) | **20.51** |  |  |

Note:

Bias-corrected bootstrap 95% CIs, bootstrapping based on 200 replications.

The proportion mediated was calculated using the formula: {HRNDE (HRNIE− 1)/(HRNDE * HRNIE− 1)}*100.

Models adjusted for maternal education, social class and maternal age at birth, previous live births (and cohort in pooled sample).
